# Energy, hierarchy and the origin of inequality

**DOI:** 10.1371/journal.pone.0215692

**Published:** 2019-04-24

**Authors:** Blair Fix

**Affiliations:** York University, Toronto, Ontario, Canada; The Bucharest University of Economic Studies, ROMANIA

## Abstract

Where should we look to understand the origin of inequality? I propose an unusual window of evidence—*modern societies*. I hypothesize that evidence for the origin of inequality is encoded in the institutional structure of industrial societies. To test this idea, I use a model to project modern trends into the past. This model takes the modern relation between energy, hierarchy, and inequality and creates a hindcast of the origin of inequality. The results are broadly consistent with the available evidence. The model predicts an explosion of inequality with the transition from hunter-gathering to agriculture, followed by a plateau. This finding potentially opens a new window of evidence into the origin of inequality.

## 1 Introduction

The origin of inequality is one of the great mysteries of human social evolution. For the vast majority of our history, we lived in small bands that were fiercely egalitarian [1]. But then around 10,000 years ago, something changed [2, 3]. For reasons that remain poorly understood, we began to abandon our ancestral state, and started allowing some individuals to command vastly more resources than others. At first inequality was the exception, but it soon spread until it became the most common form of organization.

This great transition has puzzled scientists for centuries [2–11]. But like the origin of life, the origin of inequality is frustratingly difficult to study. The problem is that origins remain locked in the past, meaning evidence is sparse. Still, we have made progress. With great effort, we have found three ‘windows’ of evidence into the origin of inequality: the archaeological record [10–19], surviving traditional societies [20–25], and the written record of inequality [26–30].

These windows focus either on societies that are long gone, or societies whose form is archaic. This is perfectly reasonable, but it also limits the evidence we can uncover. The archaeological and written record of inequality will always be sparse. And traditional societies are rapidly disappearing from the world. Given the limits of these windows, where else might we look to study the origin of inequality? I suggest we draw inspiration from evolutionary biology.

One of the great breakthroughs in studying the origin of life was the discovery that the DNA of living organisms contains a coded history of their evolution [31]. Might something similar be true of human societies? Might the social structure of modern societies contain a coded history of the origin of inequality? I test this possibility here. I use *institutions* as the social corollary of DNA. Institutions are systems of organizing that are passed between generations. I think we can use modern institutional trends to infer the origin of inequality.

Looking at modern societies, I find two important trends (Section 2). First, societies that use more energy tend to have *larger* institutions. Second, modern institutions are *hierarchically* organized and income increases rapidly with rank. How does this relate to the origin of inequality? The key is that the growth of institution size can be interpreted as the growth of *hierarchy*. The idea is that as hierarchy grows it concentrates resources at the top, potentially leading to greater inequality. The modern trend is towards *greater* energy use and *greater* hierarchy. To infer the origin of inequality, I propose that we *reverse* this trend and project it backwards in time. I call this the ‘energy-hierarchy-inequality’ (EHI) hypothesis:

### Energy-hierarchy-inequality hypothesis

We can infer the origin of inequality from the modern relation between energy use, hierarchy, and inequality.

Like with DNA, these institutional trends do not give *direct* evidence of our past. Instead, they must be interpreted with a model. To test the EHI hypothesis, I use a model to project modern trends into the past (Section 3). The model gives a hindcast of the origin of inequality—a prediction that can be compared to empirical evidence. The results are promising (Section 4). Consistent with the available evidence, the model predicts an explosion of inequality during the energy transition from hunter-gathering to agriculture. As energy use increases beyond agrarian levels, the model predicts that inequality should plateau. Whether this plateau is consistent with evidence is less clear. Depending on the inequality metric used, there is evidence that inequality *declines* slightly with industrialization. This may be because hierarchies become less ‘despotic’ as energy use increases. Future research is needed to test this possibility.

The results suggest that institutional trends in modern societies provide a plausible window into the origin of inequality. I speculate about causal mechanisms in Section 5, but for now the evidence is too sparse to draw many conclusions. More importantly, this finding opens new doors for *future* research. It implies that looking to the past may not be the only way to understand the origin of inequality. Signs of humanity’s deep history may be encoded in the institutional structure of our own societies.

## 2 Energy, hierarchy, and inequality: The evidence

I review here the evidence linking energy, hierarchy, and inequality. The chain of reasoning (but not necessarily causation) is:

energy⟶institutionsize⟶hierarchy⟶power⟶income

I begin with *energy* because, like many scientists [32–41], I think social evolution is tied to energy use. The rationale is simple: according to the laws of thermodynamics, a non-equilibrium system must be supported by a flow of energy [42]. Since human societies are non-equilibrium systems, energy should play an important role in social evolution.

The link between energy and *inequality* has been proposed before [43–46], but this paper makes two new contributions. First, I explicitly link energy and inequality through social hierarchy. Second, I develop a formal model that hindcasts the origin of inequality.

### 2.1 Energy and institution size

**Fig 1:**
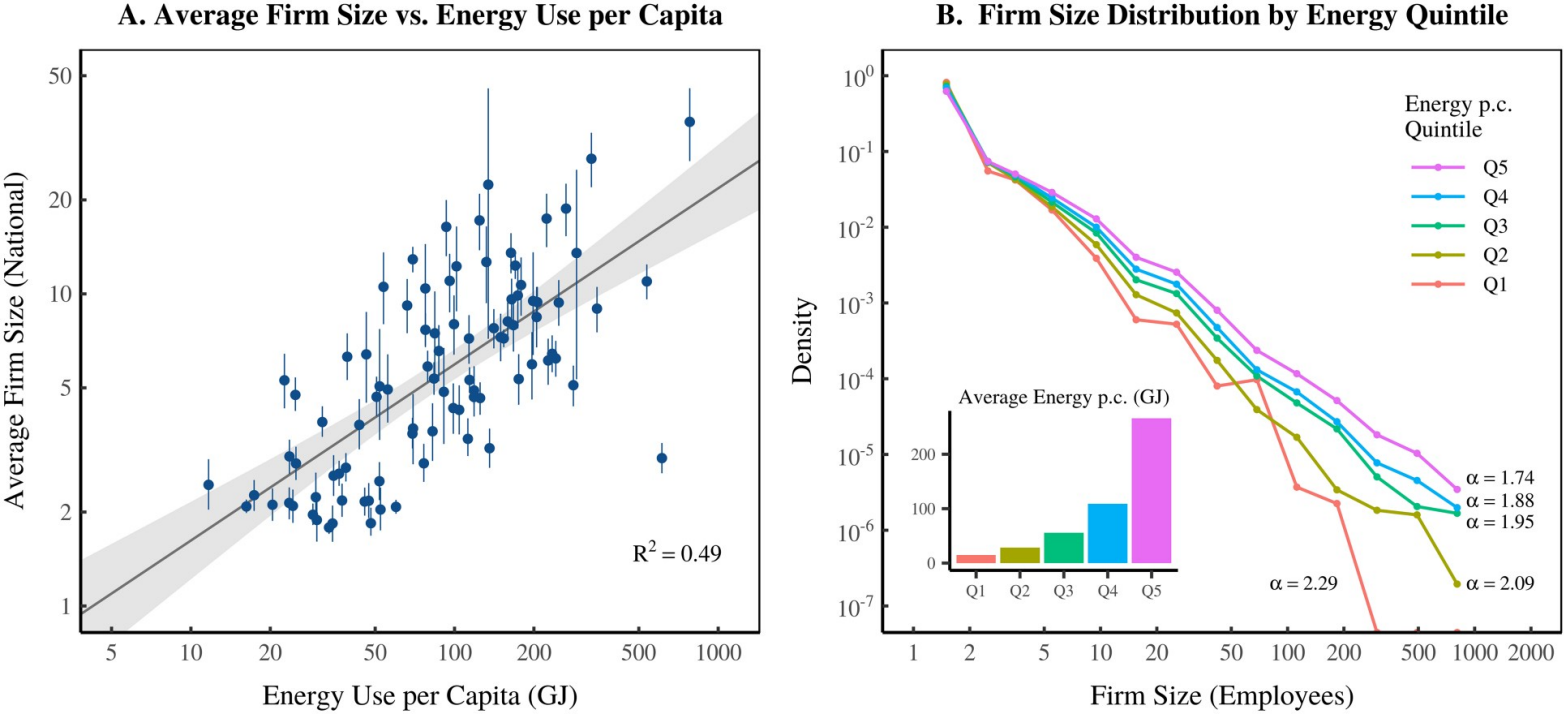
How firm size changes with energy use per capita. Panel A shows how average firm size within nations varies with energy use per capita. Firm size is measured using employment. Each data point represents a country. Error bars indicate the 95% confidence interval in the estimates of mean firm size. Grey regions indicate the 95% confidence region of the regression. Panel B shows how the entire firm size distribution within nations varies by energy use. I put countries into 5 groups, ranked by energy use. I then plot the aggregate firm size distribution within each group. The inset graph shows average energy use per capita within each quintile. Here *α* refers to the estimated power-law exponent of the firm size distribution. For sources and methods, see Section 7.

The energy-hierarchy-inequality hypothesis begins with a link between energy and institution size. In modern societies, institution size is strongly correlated with energy use per capita [47, 48]. Fig 1 illustrates this effect using business firms. Fig 1A plots average firm size within different nations against their energy use per capita. Each point represents a country, with error bars indicating the uncertainty in average firm size. As energy use per capita increases, average firm size increases as well.

The growth of average firm size is *not* caused by a horizontal shift in the distribution. Instead, it is caused by a *fattening* of the distribution tail. Fig 1B visualizes this behavior. Here I group the countries of the world into quintiles (5 groups) ranked by energy use per capita. For each quintile, I plot the aggregate firm size distribution. Note how the slope of the firm size distribution *decreases* with greater energy use. This indicates that large firms become more common.

The firm size distribution can be modeled by a power law [48–51]. This means that the probability of finding a firm of size *x* is roughly proportional to *x*^−*α*^, where *α* is the power-law exponent. A *smaller* power-law exponent indicates a *fatter* tail. As shown in Fig 1B, greater energy use is associated with a *smaller* power-law exponent for the firm size distribution. This provides a simple way to model the relation between energy use and firm size.

### 2.2 Institution size and hierarchy

The second step of the energy-hierarchy-inequality hypothesis is to connect institution size to hierarchy. I hypothesize that (virtually) all human institutions are hierarchically organized. This means they have a nested chain of command that grows with institution size. As the hierarchy grows, new ranks are added at a logarithmic rate [52, 53]. This scaling behavior has been observed in business firms [54], historical empires [55], and hunter-gather societies [56]. Hierarchical organization also means that elite ranks should become more common as a hierarchy grows. Assuming that *managers* occupy top ranks, this implies that the management share of employment should increase with average firm size. This trend has been observed at the international level [48].

**Fig 2:**
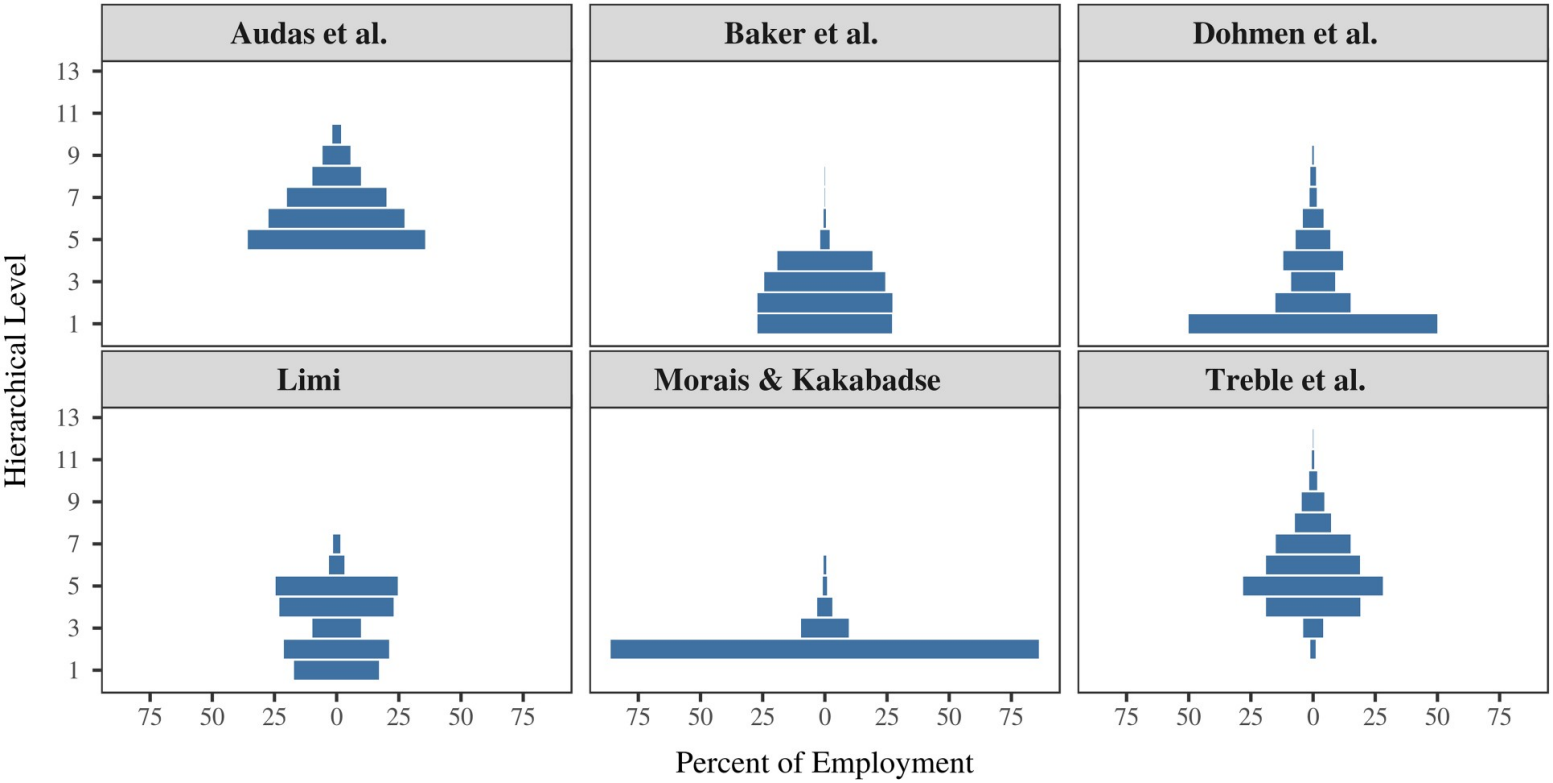
Hierarchical employment structure of six case-study firms. This figure shows the hierarchical employment structure of six different case-study firms, named after the study authors [57–62].

The most direct evidence for hierarchical organization comes from firm case studies [57–62]. Fig 2 shows the hierarchical structure of six case-study firms (which come from Britain, the Netherlands, Portugal, and the United States). Although the specific structure varies, all six firms share the pyramid shape that we expect of a hierarchy. I use these case studies to inform the energy-hierarchy-inequality model (see Section 7 for details).

To summarize, the evidence suggests that institutions tend to become larger as energy use increases. If institutions are hierarchically organized, this implies that the growth of energy is associated with the growth of hierarchy.

### 2.3 Hierarchical power and income

The last component of the energy-hierarchy-inequality hypothesis is a relation between hierarchical power and income. The idea is that elites use their power within a hierarchy to gain preferential access to resources.

Why might this be the case? Our evolutionary background provides some hints. Virtually all social mammals form dominance hierarchies [63–68]. In these hierarchies, high social status allows greater access to resources, particularly sexual mates [69–74]. Given our evolutionary heritage, we expect that humans should exhibit similar behavior. Unsurprisingly, there is a strong link between human hierarchical status and reproductive success [75–79].

**Fig 3:**
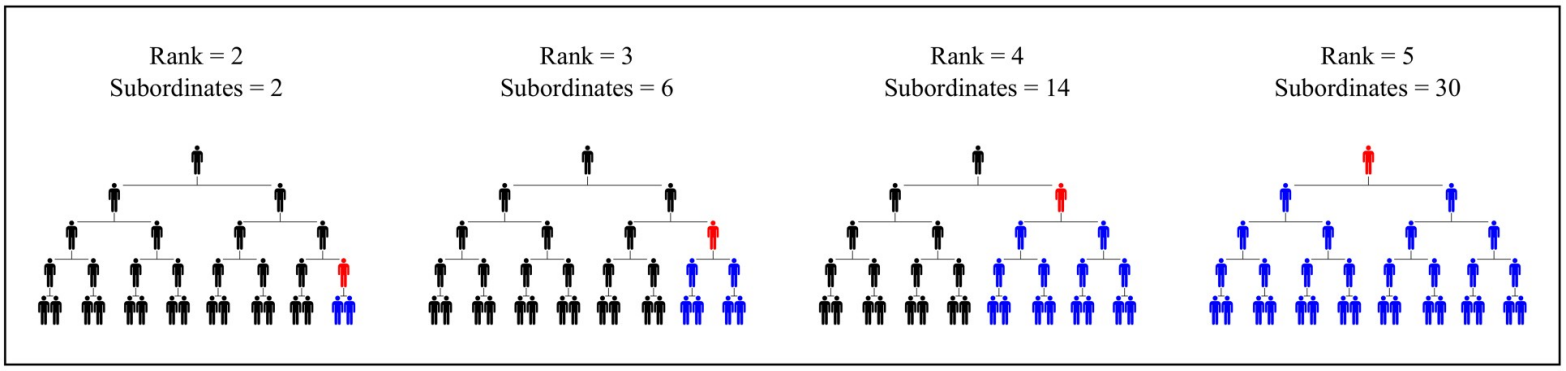
The exponential growth of subordinates with rank. In an idealized hierarchy, the total number of subordinates (blue) tends to grow exponentially with hierarchical rank (red). The exact relation will depend on the *span of control*—the number of subordinates directly below each superior.

Is the same true for income? Evidence suggests so. But before looking at this evidence, I note a key difference between human and non-human hierarchies. All other animals form *linear* hierarchies—an ordinal ranking from top to bottom. But humans form *branching* hierarchies, in which each superior controls *multiple* subordinates. This has important consequences for income distribution. In a branching hierarchy, the number of subordinates grows *exponentially* with rank (Fig 3). If income stems from power over subordinates, than it too should increase exponentially with rank. This means that hierarchy can lead to vast inequalities.

To make this relation quantitative, I define ‘hierarchical power’ as:

hierarchicalpower=1+numberofsubordinates (1)

The idea is that control over subordinates is a form of power—it increases “the possibility of imposing one’s will upon the behavior of other persons” [80]. All individuals start with a baseline power of 1, indicating they have control over themselves. Hierarchical power then increases proportionally with the number of subordinates.

**Fig 4:**
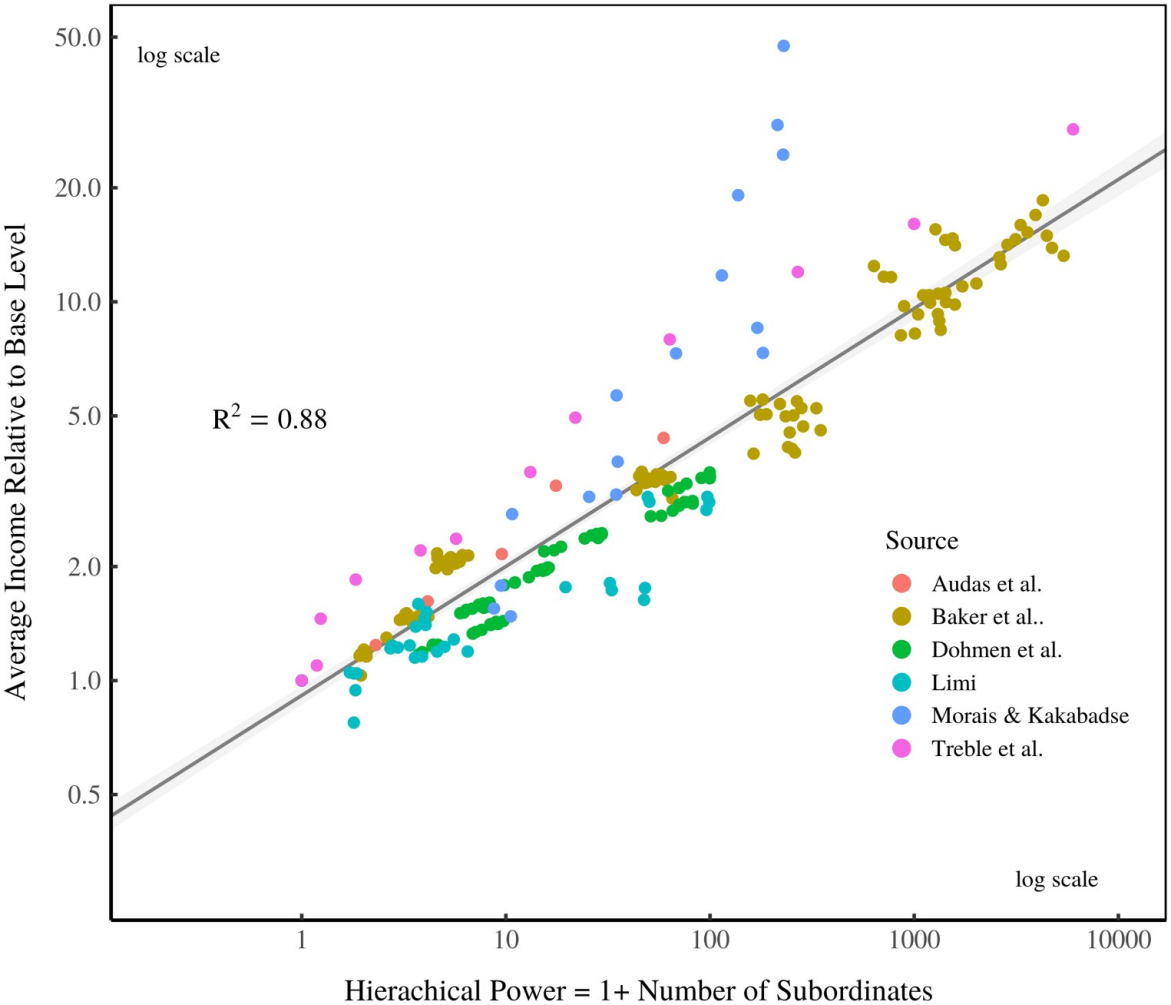
Average income vs. hierarchical power within case-study firms. This figure shows data from six firm case studies [57–62]. The vertical axis shows average income within each hierarchical level of the firm (relative to the base level), while the horizontal axis shows my metric for average power, which is equal to one plus the average number of subordinates below a given hierarchical level. Each point represents a single firm-year observation, and color indicates the particular case study. Grey regions around the regression indicate the 95% prediction interval. For methods, see Section 7.

Is income within hierarchies a function of hierarchical power? Evidence from case-study firms suggests so. Fig 4 plots average income (relative to the bottom hierarchical level) against average hierarchical power for each rank in our six case-study firms. There is a strong correlation. A similar correlation exists between *changes* in income and *changes* in hierarchical power [81].

**Fig 5:**
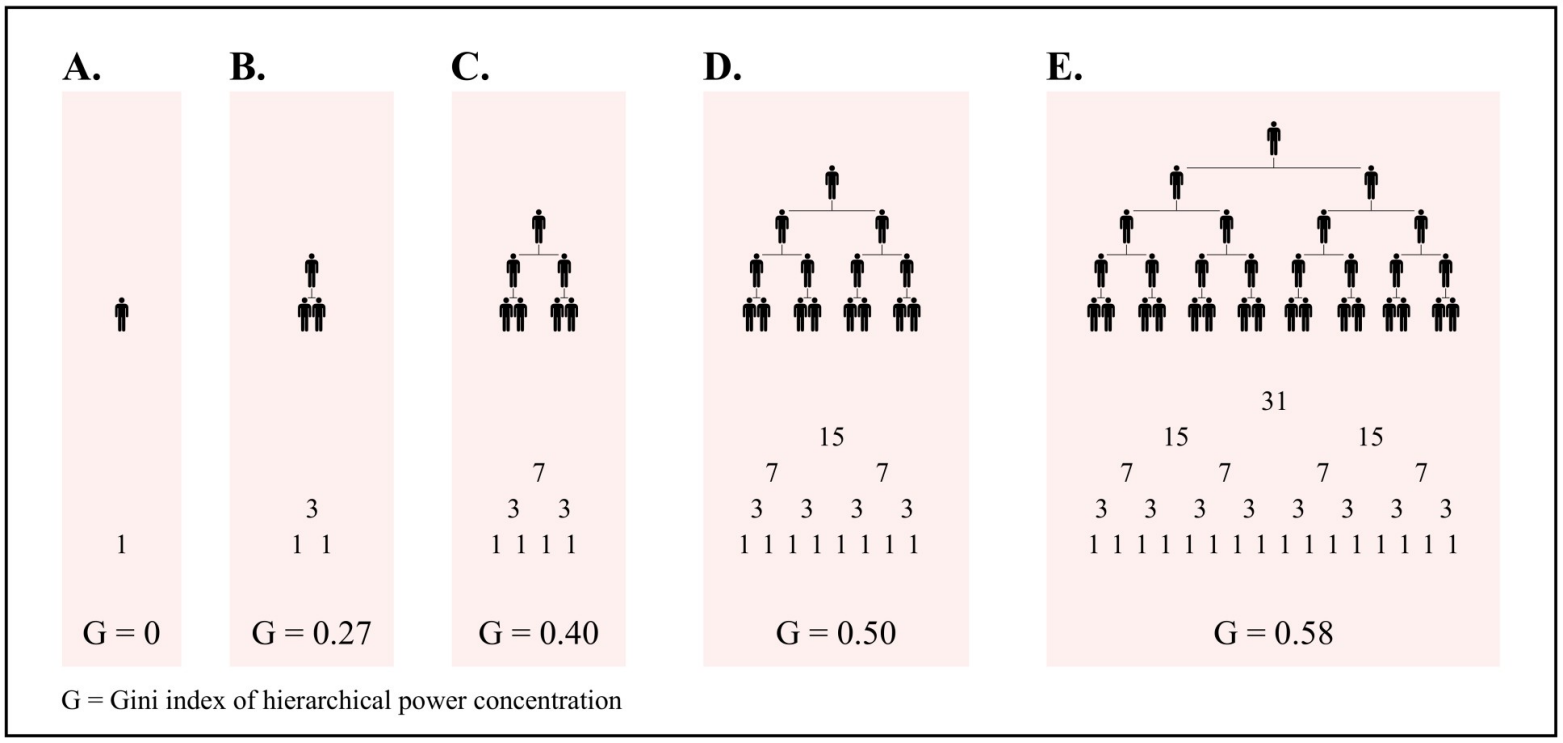
The growth of hierarchy concentrates power. This figure illustrates how the growth of hierarchy leads to the concentration of hierarchical power. Below each hierarchy, I show the distribution of hierarchical power. (hierarchical power = 1 + the total number of subordinates). I then calculate the Gini index of hierarchical power concentration (G). The initial growth of hierarchy rapidly concentrates power. But further growth of hierarchy leads to progressively slower growth of hierarchical-power concentration.

The power-income relation implies that inequality should increase as a hierarchy grows. This is because hierarchical power gets concentrated as a hierarchy gets larger (Fig 5). Importantly, this relation is *non-linear*. The initial growth of hierarchy rapidly concentrates power. But further growth of hierarchy leads to progressively slower growth of hierarchical-power concentration. If income scales with hierarchical power, the same should be true of inequality. As a hierarchy grows, inequality should explode and then plateau.

To summarize, modern evidence suggests a joint relation between energy use, hierarchy, and inequality. As energy use increases, societies become more hierarchical. If income is proportional to hierarchical power, this should cause an increase in income inequality. To investigate the origin of inequality, I propose that we extrapolate this relation back in time.

## 3 An energy-hierarchy-inequality model

To extrapolate the energy-hierarchy-inequality evidence, I create a numerical model. This model simulates the empirical relation between energy, hierarchy, and income. I discuss the basic components of the model below. For a technical discussion, see Section 7.

### 3.1 Model assumptions

The energy-hierarchy-inequality model extrapolates modern trends into the distant past. To do this, we assume the following:

Assumption 1 Institutions have a power-law size distribution. The growth of institution size is synonymous with a decline in the power-law exponent.
Assumption 2 Institutions are hierarchically organized with a structure equivalent to modern firm hierarchies.
Assumption 3 The modern trend between energy use per capita and institution size applies to all societies.
Assumption 4 Income scales with hierarchical power in all societies. The rate of scaling may vary over time and space.

Are these assumptions realistic? Regarding assumption 1, there is evidence that pre-capitalist societies had a power-law distribution of institution size. For instance, feudal manor size was roughly power-law distributed [82, 83]. Similarly, slave estate size in the antebellum American South was roughly power-law distributed (see S1 Fig). Evidence also suggests that hunter-gatherer settlement sizes had a power-law distribution tail [84]. The *types* of institution certainly vary across time and space. But regardless of type, the power-law distribution of institution size seems common.

Assumptions 2, 3 and 4 are speculative. But given empirical evidence, why not extrapolate it and see where it takes us?

### 3.2 Model structure

The energy-hierarchy-inequality model has four main steps, discussed below. For technical details, see Section 7.

**Step 1: Generate the institution-size distribution**. The model generates an institution size distribution using a discrete power law. The power-law exponent varies stochastically over different model iterations. This simulates changes in institution size.
**Step 2: Estimate energy use from institution size**. Energy use per capita (*E*_*pc*_) is modeled as a function of average institution size $\bar{I}$:
  $$E_{pc}=c_{1}{\bar{I}}^{c_{2}}$$ (2)
  The parameters *c*_1_ and *c*_2_ are determined from a regression on the international energy and firm data shown in Fig 1A.
**Step 3: Create hierarchical structure**. The model uses firm case-study data (Fig 2) to determine the hierarchical structure of institutions. All modeled institutions have the same ‘shape’, but the number of ranks varies with institution size.
**Step 4: Endow individuals with income** Individual income *I* scales with hierarchical power *P* as
  $$I\propto P^{\beta }\cdot \epsilon$$ (3)
  where *β* determines the rate of scaling and *ϵ* is a noise factor. To simulate variation between societies, *β* varies stochastically between model iterations. I use case studies of modern firms, as well as an antebellum US slave estate, to determine a plausible range for this variation. The noise factor *ϵ* adds a small amount of dispersion to the power-income relation. This is determined by income dispersion within hierarchical levels of the case-study firms. On its own, the noise factor corresponds to a Gini index of about 0.1.
**Between-Institution Income Dispersion**. The model *excludes* income dispersion between institutions. US evidence suggests that between-institution income dispersion accounts for a minority of total income dispersion (about 30%) [85]. I assume that the growth of between-institution dispersion is not important for the emergence of inequality. Future research can determine if this is an appropriate assumption.

### 3.3 Visualizing the energy-hierarchy-inequality model

**Fig 6:**
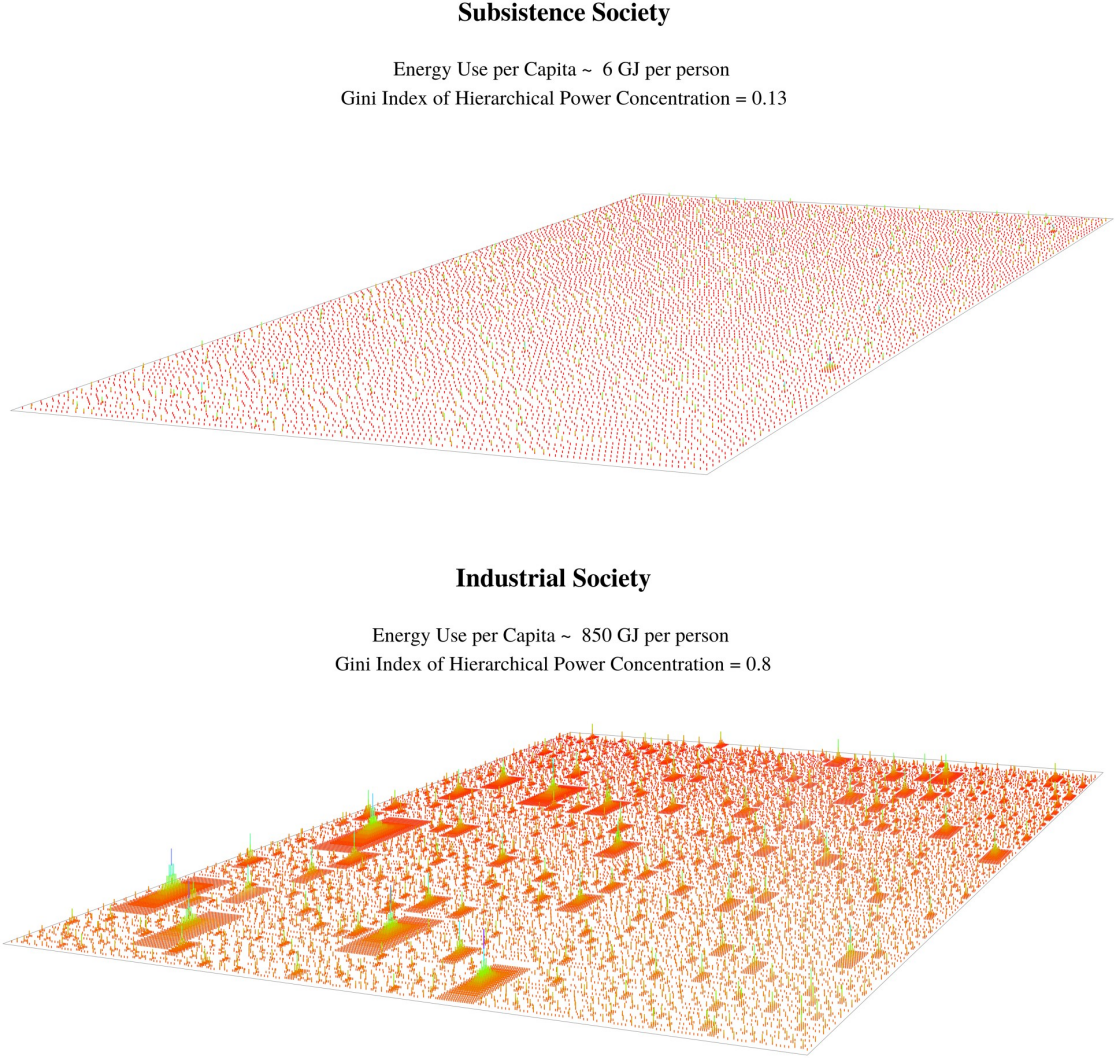
Visualizing the energy-hierarchy-inequality model. This figure shows the EHI model as a landscape. Hierarchies are visualized as pyramids. Height and color indicate hierarchical rank. The top panel shows a subsistence society that consumes hunter-gatherer levels of energy use. The model predicts little hierarchical organization, and little concentration of hierarchical power. The bottom panel shows an industrial society with energy use on par with modern Iceland or Qatar. The model predicts considerable hierarchical organization, and considerable concentration of hierarchical power.

Fig 6 visualizes the energy-hierarchy-inequality model as a landscape. Hierarchies appear as pyramids, with hierarchical rank indicated by height and color. On top is a subsistence society that consumes 5GJ of energy per capita per year. This is 3200 Kcal per day—not much above the metabolic needs of an average person. Hierarchical organization is negligible. Consequently, hierarchical power is very equally distributed, with a Gini index of 0.13. We expect very little inequality in this society.

On the bottom is an industrial society that consumes 500GJ of energy per capita per year—similar to modern Iceland or Qatar. Hierarchical organization is ubiquitous. Consequently, hierarchical power is extremely concentrated, with a Gini index of 0.76. We expect significant inequality in this society.

## 4 Extrapolating the origin of inequality

**Fig 7:**
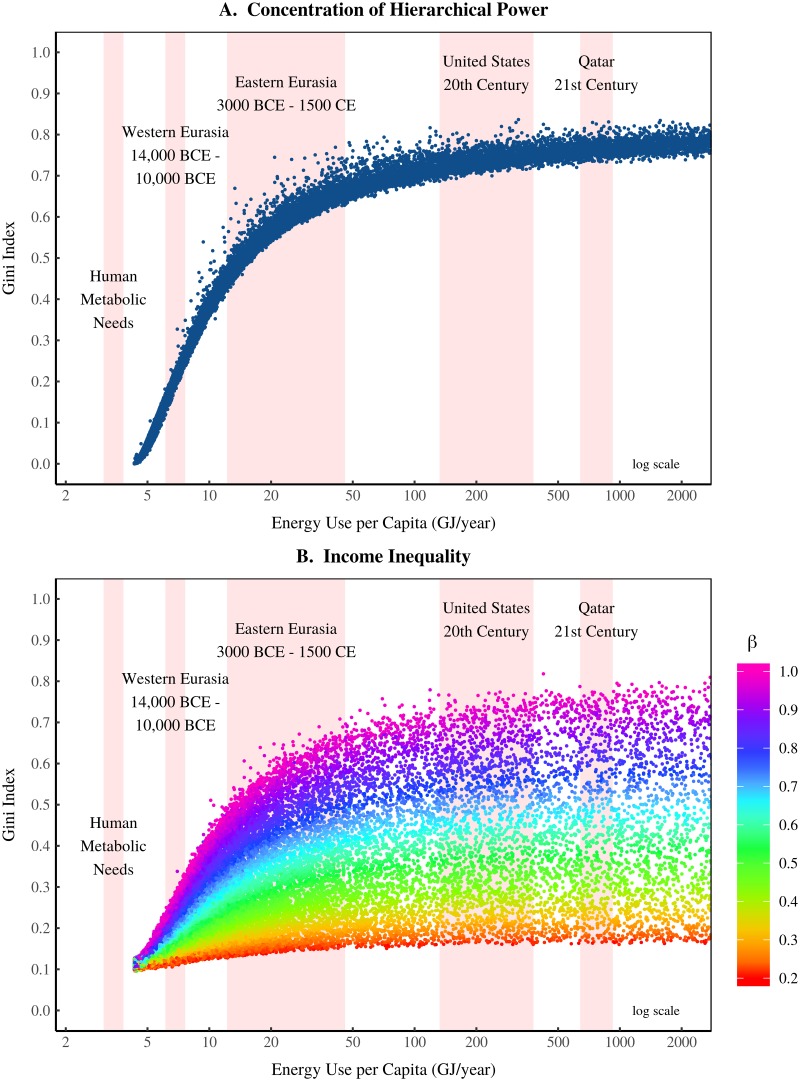
Extrapolating the origin of inequality with the EHI model. This figure shows the results of the energy-hierarchy-inequality model. Panel A shows how the concentration of hierarchical power changes with energy use per capita. Panel B shows the evolution of income inequality. Color indicates the scaling exponent *β* between hierarchical power and income (see Eq 3). Shaded regions show the energy use range for various societies throughout history. For sources and methods, see Section 7.

I use the EHI model to extrapolate the origin of inequality. Fig 7 shows the predicted relation between energy use, the concentration of hierarchical power, and inequality. There are four notable predictions:

1. **Hierarchy vanishes at metabolic levels of energy use, causing a collapse of inequality**. Hierarchical organization disappears as energy use approaches metabolic levels (i.e. food energy only). Consequently, hierarchical-power concentration is eliminated and inequality becomes negligible.
2. **Inequality explodes during the transition to agriculture**. Virtually all increases in inequality occur during the transition from subsistence to agrarian levels of energy use. (In Fig 7, agrarian energy use is represented by Eastern Eurasia from 5,000 BCE to 1500 CE [86]).
3. **The range of inequality grows with energy use**. The transition to agriculture opens a huge range of ‘inequality space’. The governing factor is *β*—the rate that income scales with hierarchical power. Societies with low *β* remain equal during the transition to agriculture. But societies with high *β* experience an explosion of inequality.
4. **Energy growth beyond agrarian levels has little effect on inequality**. After the transition to agriculture, the concentration of hierarchical power plateaus. As a result, further increases in energy use have a negligible effect on inequality.

### 4.1 Testing the energy-hierarchy-inequality prediction

**Fig 8:**
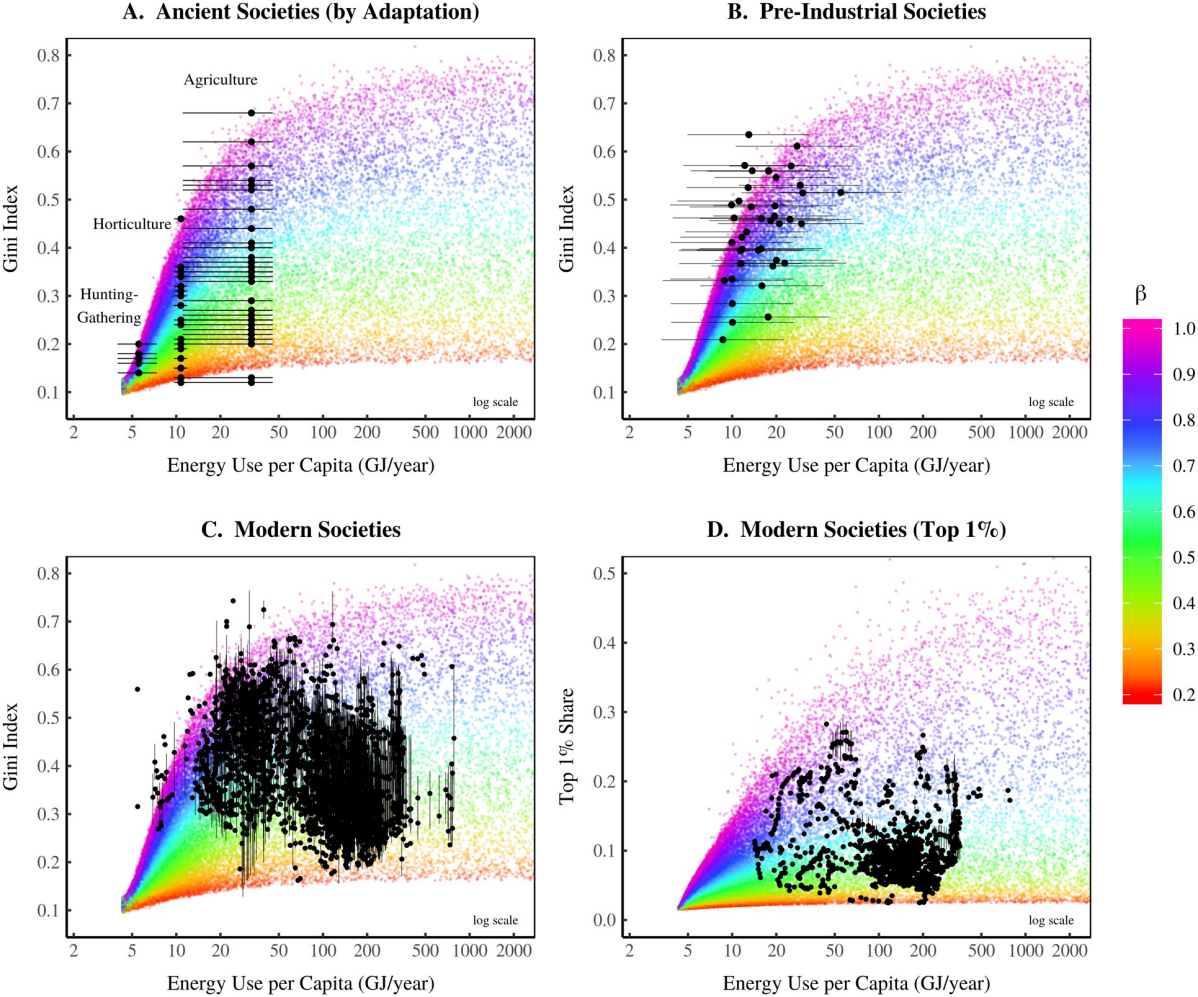
Testing the energy-hierarchy-inequality model. This figure compares the EHI model to empirical data. Panel A shows archaeological data from ancient societies, measured using housing size and reported by ‘adaptation’. Horizontal lines indicate the plausible range of energy use for each adaptation. Panel B shows income inequality in pre-industrial societies. Energy use is estimated from per capita income data (horizontal lines show the uncertainty). Panel C shows data for modern nation-states, with vertical lines showing the range of inequality estimates for each country. Panel D also shows modern data, but measures inequality using the top 1% income share. For sources and methods, see Section 7.

The EHI model predicts how the emergence and evolution of inequality should relate to energy use. Fig 8 compares this prediction to the available evidence.

Fig 8A compares the model to archaeological data for ancient societies. The caveat is that the archaeological data measures inequality using house size [18]. This is not strictly comparable to the ‘income inequality’ produced by the EHI model. Nonetheless I make a comparison. The archaeological data is grouped by societal adaptation. Horizontal error bars indicate the plausible range of energy use for each adaptation. Points represent the mean estimate. (For sources and methods, see Section 7). The model’s prediction is consistent with the archaeological evidence—inequality explodes during the transition to agriculture.

Fig 8B compares the model to data from pre-industrial societies [29]. Horizontal error bars show the uncertainty in energy use (which is estimated from GDP). Again, the model is consistent with the empirical data. In pre-industrial societies, inequality increases rapidly with energy use.

Fig 8C compares the model to modern evidence. The model’s range is consistent with the empirical data. But there is a downward trend in the empirical data that is not predicted by the model. I discuss possible interpretations of this trend below. Fig 8D also compares the model to modern evidence, but measures inequality using the top 1% income share. The empirical data is in a range that is consistent with the model. Again, there is a downward trend in the empirical data, but far less pronounced than in Fig 8C.

To summarize, EHI model predictions for the origin of inequality are consistent with the available evidence. But for industrial societies, the model predictions are more ambiguous. Modern evidence is within the range predicted by the model. However, the data shows a decline of inequality with energy use that is not predicted.

### 4.2 The Kuznets curve: The decline of hierarchical despotism?

**Fig 9:**
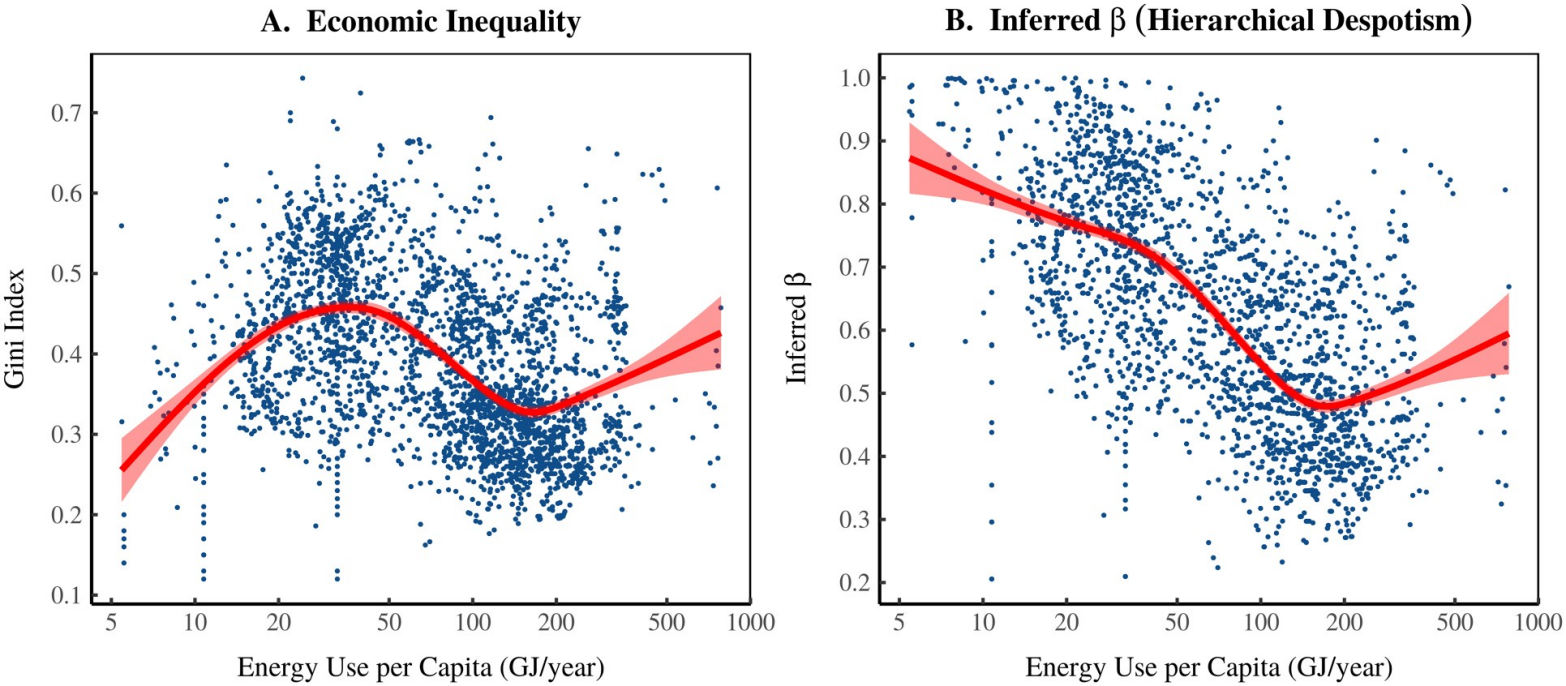
Is the Kuznets curve caused by declining hierarchical despotism? Panel A plots all of the empirical data in Fig 8A–8C. The red line shows the smoothed trend. It has an inverted U shape, often called a ‘Kuznets curve’. Panel B shows inferred *β* for each society. This is the scaling of income with hierarchical power that is required if the EHI model is correct. I infer *β* by matching real-world societies to the EHI model. I interpret *β* as an index of ‘hierarchical despotism’—it measures elites’ ability to use their hierarchical power to concentrate resources.

Fig 9A aggregates all the empirical data in Fig 8A–8C to show the long-term trend between energy use and inequality. A Kuznets curve-like pattern [87] emerges (an inverted U-shaped relation). Inequality tends first to increase with energy use, and then decline. The increase is predicted by the model, but the decrease is not. Is the model wrong?

More evidence is required to answer this question. The problem is that the model predicts a huge range of ‘inequality space’ for industrial societies. The range of this space is determined by *β*—the scaling of income with hierarchical rank. I have assumed that the distribution of *β* is *independent* of energy use. But this could be wrong. To test the model, we need independent estimates of *β* in real-world societies. Such estimates do not presently exist.

While we cannot confirm or falsify the model, we can infer how *β* *should* behave if the model is correct. To do this, we match the empirical data to the best-fit model iteration. We then assign the model’s *β* to the real-world society. The resulting inference is shown in Fig 9B. If the model is correct, *β* should *decline* with energy use.

Future research can test this inference. For now, I reflect on what it means. The parameter *β* determines how rapidly income scales with hierarchical power. I interpret *β* as an index of *hierarchical despotism*. It measures elites’ ability to use their hierarchical power to concentrate resources. A larger *β* indicates a more despotic hierarchy (greater returns to hierarchical power). The model predicts that hierarchical despotism declines as energy use increases.

This suggests that the Kuznets curve is created by two trends that accompany increases in energy: (1) the growth of hierarchy; and (2) the decline of hierarchical despotism. The first half of the Kuznets curve is created by the growth of hierarchy, which concentrates hierarchical power, leading to greater inequality. But hierarchical power concentration eventually plateaus. At this point, the decline in hierarchical despotism dominates the trend. This causes the second half of the Kuznets curve—inequality declines with greater energy use.

The decline of hierarchical despotism is an untested inference. But it seems plausible. History suggests that as societies develop, they introduce checks on power. These include the rule of law, democracy, and labor unions. Might these checks on power gradually reduce hierarchical despotism? Future research can test if this is true.

## 5 Discussion

The results of the model suggest that the energy-hierarchy-inequality hypothesis is plausible. By projecting modern trends into the past, we can accurately predict the origin of inequality. I discuss here some of the implications. I begin with the model’s limitations, and then speculate about causation and the role of hierarchy in the emergence of inequality.

### 5.1 Model limitations

The energy-hierarchy-inequality model is built on *correlation* and is not intended to answer questions of *causation*. This is a limitation, but is also the main reason the model provides insight.

The model takes two correlations as inputs: (1) the correlation between energy use and institution size; and (2) the correlation between hierarchical power and income. The model does not explain why these correlations *exist*. Instead, it explains why they might be *important* for the origin of inequality. The model indicates that *if* these trends existed in the past, they *imply* that inequality arose during the transition to agriculture.

Even though this operates at the level of correlation, it is an important insight. It suggests that modern trends provide a new window into the origin of inequality. The task for future research is to use this window to better understand the ultimate causes.

### 5.2 Causation

For the EHI hypothesis, understanding causation means explaining our two correlations. We want to know *why* the growth of energy use relates to the growth of hierarchy, and *why* income increases with hierarchical power. Answering these questions exceeds the scope of this paper. But I will speculate here.

I suspect that energy relates to hierarchy via a feedback loop, meaning causation runs both ways. I think this because different evidence suggests different causal directions. The collapse of the Soviet Union is one example where *hierarchy* seems to drive *energy* use. When the Soviet government collapsed, energy use in former Soviet states decreased dramatically [48]. Because there was no global energy shortage at the time, we can plausibly infer that the institutional collapse *caused* the decline in energy use.

But we can also think of reasons for the *reverse* causation—when *energy* drives (or limits) the growth of *hierarchy*. This ties into the surplus theory of social stratification [88–90]. In agrarian societies, the energy surplus of farmers is too small to support many non-farm workers [34]. Thus, there is little room for a managerial class. But if the surplus grew, it could loosen these limits and allow the growth of hierarchy [48, 91]. This suggests that energy growth could cause the growth of hierarchy.

These two examples suggest that causation can run both ways—energy can drive the growth of hierarchy and vice versa. Untangling this causal process is a difficult task for future research.

What causes the relation between income and hierarchical power? I think it is likely caused by many different factors. In despotic hierarchies (such as slave plantations), superiors may use coercion and brute force to derive their income. But in less despotic hierarchies, *ideology* is likely more important. The substance of these ideologies differs, but the function is always the same—to justify the power of elites. Traditional societies often justify power through *kinship*—tracing lineage to a founding ancestor [92, 93]. Feudal societies use *religion*, as in the divine right of kings [94]. Capitalist societies use *ownership* to justify power [95]. In each society, the ideology justifies both the authority of elites and their greater access to resources. To understand why income relates to hierarchical power, I think we must understand the ideologies that legitimize power. These ideologies have been well studied [95–111], but much remains to be learned.

We must also take seriously the social practices that evolve to *check* the power of elites. In modern societies, this would include labor unions and democratic oversight. There is strong evidence that labor unions limit inequality [112, 113]. This suggests that by organizing low-ranked individuals, unions check the power of elites. There is also evidence that democratic oversight limits the income of elites. For instance, US CEOs in industries that are regulated by government earn less than CEOs in non-regulated industries [114]. And elite compensation in the democratically-controlled public sector is far lower than in the private sector. As one example, the US president earns about 40 times less than CEOs in the largest US firms [115–117].

While there are many plausible causes for the power-income relation, studying them brings us back to the problem of measurement. I focused on hierarchical power because it was easy to quantify. But when we try to look under the hood of this power, measurement becomes difficult. For instance, how do we measure the effect of an *ideology*? To understand causation, we must wrestle with these difficulties. Again, this is a task for future research.

### 5.3 The emergence of hierarchy

Although we poorly understand the mechanisms at work, I want to speculate about the origin story told by the EHI model. It suggests that the origin of inequality can be reframed as the emergence of *hierarchy*. But this raises a question. After hundreds of thousands of years of living in (relatively) egalitarian societies, why would humans suddenly choose to organize in despotic hierarchies?

Scientists have long puzzled over this question. Was there an advantage to hierarchy, as functionalist theory contends [118, 119]? Or was it a matter of coercion, as conflict theory contends [102, 120–122]? Or did the emergence of hierarchy involve both function and coercion [101, 123–125]? I think the latter is most likely. Without a functional advantage, it is hard to understand why hierarchy would emerge. But without coercion, it is hard to understand the great inequalities that exist within hierarchies.

Let’s begin with the advantages of hierarchy. The modern evidence indicates that hierarchy increases with energy use. One interpretation is that hierarchy somehow enables, or is necessary for, greater energy use (for a different interpretation, see [91]). If this is true, then we need to ask two questions. First, why is using more energy advantageous? Second, why is hierarchy required to use more energy?

Regarding the first question, if life is the struggle for energy [35, 126], then using more energy may give a competitive advantage to an organism (or group of organisms). This is the idea behind the *maximum power principle*, which attempts to give an energetic basis to Darwinian fitness [127–129]. It proposes that organisms (and ecosystems) evolve to maximize power—the flow of energy per unit of time. While it has some empirical support [130, 131], the maximum power principle remains controversial.

Still, there are clear instances where using more energy is advantageous to human groups. The most conspicuous is warfare. The evolution of military armament moves towards increasingly devastating weaponry (bows and arrows, guns, missiles, and nuclear warheads). This reduces to energetics: the destructive capability of a weapon is proportional to the amount of energy it releases. We need only look at the history of European conquests to see how better armament led to a group advantage [132]. Greater energy use may also allow reproductive benefits. For instance, in existing traditional societies, agrarian societies tend to have higher fertility than hunter-gatherers and horticulturists [133]. To summarize, using more energy may be advantageous in inter-group competition. We can think of this as a form of ‘group selection’ [134, 135]. The idea is that groups that use more energy outcompete groups that use less energy.

But why is greater energy use associated with greater *hierarchy*? One possibility is that using more energy requires greater social coordination, and hierarchy is the most potent way to achieve this. Here is my reasoning. Increasing energy use involves profound technological changes. Most notably, the scale and complexity of technology increases [48]. I suggest that this increasing complexity requires more social coordination. This is where hierarchy comes in. While humans can organize without hierarchy, the scale appears limited. The problem is that human sociability likely has biological limits [136]. Individuals generally cannot maintain more than a few hundred social relations. Hierarchy sidesteps these limits [55]. A member of a hierarchy needs to interact only with his direct superior and direct subordinates. This allows group size to grow without the need for more social interactions.

If hierarchy confers energetic benefits (via coordination), we can imagine a feedback loop emerging: Hierarchical organization enables large-scale coordination that then enables greater energy use, that then enables more hierarchy (and so on). This explains why energy and hierarchy go together. But it leads to a problem. For the vast majority of human history, hierarchical organization was negligible. Clearly there was no energy-hierarchy feedback loop. What are we missing?

The missing ingredient is resource distribution *within* the hierarchy. The problem is that hierarchy is a double-edged sword. It allows greater coordination, but it also leads to *despotism*. The nested chain of command gives enormous power to top-ranked individuals. When this power is (predictably) used for personal gain, it leads to vast inequalities. This would explain why income scales with hierarchical power. The resulting inequality means that hierarchy may not benefit low-ranking individuals. If the material gains from coordination are monopolized by elites, low-ranking individuals may be better off leaving the hierarchy. The stability of a hierarchy thus depends on the net advantage for low-ranking individuals [125]. If there is no advantage, the hierarchy will be unstable.

For the majority of human history, the costs of hierarchical despotism likely outweighed any coordination benefits from hierarchy. We know that modern hunter-gatherers (and presumably ancient ones as well) aggressively suppress individuals with power-seeking tendencies [137, 138]. Without a concentrated energy source (such as agriculture) the benefits to large-scale coordination were likely marginal. Therefore, hierarchy was not tolerated because it conferred no advantage.

This likely changed during the Neolithic revolution. The details remain poorly understood, but we can guess that the benefits of large-scale coordination increased. This is likely related to sedentism and the development of agriculture [139, 140]. Irrigation likely also played an important role [141, 142]. I argue that during the Neolithic revolution, the energy-hierarchy feedback loop took hold. As a result, hierarchical power became more concentrated. Elites predictably used their power for personal gain, resulting in the emergence of inequality.

I have so far treated inequality as an *effect* of hierarchy. But it may actually play a role in the growth of hierarchy. I have argued that the growth of hierarchy depends on the net advantage to low-ranking individuals. One way to increase this advantage is to increase the returns to hierarchical coordination (through environmental or technological change). But another way to increase the net advantage is to *decrease* hierarchical despotism. If the gains of hierarchy are more equally distributed, the net benefit to low-ranking members is greater.

This reasoning means that inequality may play a causal role in the growth of hierarchy and the growth of energy use. This is speculation, but it fits with the inference that hierarchical despotism declines with energy use (Fig 9B). Perhaps limiting hierarchical despotism is a prerequisite for industrialization? Or put another way, is it possible to have an industrial economy built on slavery—the most despotic mode of human organization? These are open questions worth investigating.

To summarize, I think that understanding the energy-hierarchy-inequality relation requires merging both functional and conflict theories of social stratification. It requires understanding what Wilson calls the “fundamental problem of social life” [134]. The idea is that cooperative groups beat uncooperative groups. But selfish individuals beat unselfish individuals *within* groups. Hierarchy nicely highlights both aspects of this problem. It is a powerful tool for coordination, and thus has potential group benefits. But it is also predictably used for selfish gain, thus resulting in great inequality. Thinking in this way may provide an important tool for understanding the origin of inequality.

## 6 Conclusions

Origin questions are some of the most seductive in science. At the same time, they are among the most difficult questions to answer. The problem is that origins are always locked in the past, meaning evidence is frustratingly sparse. Scientific progress on origin questions happens when we find reliable windows into the past.

It is instructive to see how new windows of evidence have advanced other fields. In modern cosmology, the breakthrough came when Edwin Hubble discovered that galaxies are receding from us. *Reversing* this trend implied that the universe had once been smaller—perhaps infinitely so. And so the big bang theory was born [143]. In biology, the breakthrough came with the discovery of DNA. By comparing the DNA of different organisms, we can infer the history of evolution. It suggests that all life has a single origin [31].

What about the origin of inequality? Obviously we should continue to gather historical and archaeological evidence. But this evidence will always remain limited. We should also continue studying traditional societies. But these societies are rapidly disappearing from the world. That leaves modern societies as a source of evidence.

I have proposed that the institutional structure of modern societies contains a coded history of the origin of inequality. To test this idea, I used a model to project into the past the modern relation between energy use, hierarchy, and inequality. The model predictions are generally consistent with the evidence. This suggests we may have found a new window into the origin of inequality.

## 7 Methods

### 7.1 Data sources and methods

#### Sources for Fig 1

Data for firm size comes from the Global Entrepreneurship Monitor (GEM), series ‘omnowjob’. To calculate firm size, I merge all data over the years 2001-2014. Because the GEM data over-represents large firms, I use only firms with 1000 or fewer employees. For method details, see the Appendix in Ref. [48]. Uncertainty in average firm size is estimated using the bootstrap method. Firm size distribution power-law exponents are estimated using the R PoweRlaw package [144]. Energy data comes from the World Bank, series EG.USE.PCAP.KG.OE.

#### Sources for Figs 2 and 4

Firm case-study data comes from [57–62]. For a description of this data, see the Appendix in Ref. [145]. Hierarchical power is defined as *P* = 1 + *S*, where *P* is hierarchical power and *S* is the number of subordinates. Because the case studies provide data for aggregate hierarchical structure only (not the chain of command), I calculate *average* hierarchical power, ${\bar{P}}_{h}=1+{\bar{S}}_{h}$. Here *S*_*h*_ is the average number of subordinates below level *h*. It is defined as the sum of employment (*E*) in all subordinate levels, divided by employment in the level in question: ${\bar{S}}_{h}\;=\sum_{i=1}^{h-1}E_{i}/E_{h}$. Income is normalized relative to the average income in the base hierarchical level (in the year in question).

#### Sources for Fig 7

I assume that human metabolic needs range from 2000 Kcal to 2500 Kcal per day. Western and Eastern Eurasia energy use data comes from Morris [86]. US total energy consumption is from Historical Statistics of the United States, Tables Db164-171 (1900-1948) and Energy Information Agency Table 1.3 (1949-2000). US population is from Maddison [146]. Qatar data comes from the World Bank (series EG.USE. PCAP.KG.OE).

#### Sources for Fig 8

**Fig 10:**
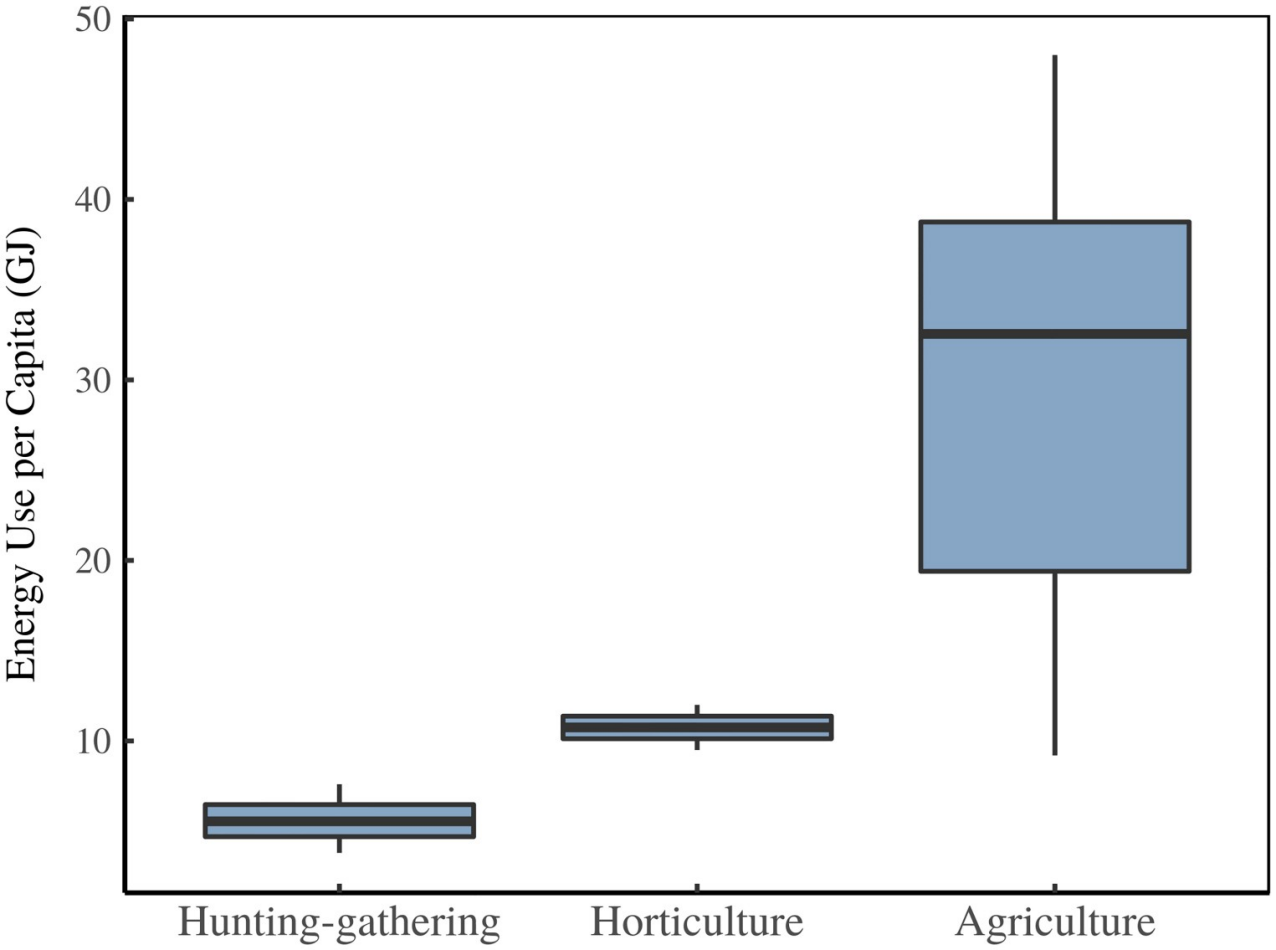
Energy use estimates by adaptation. This figure shows the energy range for historical societies sorted by adaptation. Data sources are shown in Table 1.

Fig 8A. Archaeological inequality data is from Kohler et al. [18] and is measured using house size. I estimate the energy use range for each adaptation using the data in Table 1. Results for this energy range are shown in Fig 10. Fig 8B. Pre-industrial inequality data is from Milanovic [29]. I estimate energy use from reported values of GDP per capita. To do this, I extrapolate the modern international relation between real GDP per capita and energy use per capita. Data for this regression comes from the World Bank (series EG.USE.PCAP.KG.OE and NY.GDP.PCAP.PP.KD). Fig 8C. Inequality data comes from three sources: the World Inequality Database (Gini index calculated from Lorenz curves), the United Nations World Income Inequality Database, and the OECD. I merge all data into a single database and estimate the range of inequality from this data. Energy use data comes from the World Bank, series EG.USE.PCAP.KG.OE. Fig 8D. Top 1% income share data is from the World Inequality Database. Energy use data is from the World Bank, series EG.USE.PCAP.KG.OE.

**Table 1:** Data sources for energy use by adaptation.

Society	Energy (GJ/capita)	Adaptation	Source
Agrarian max	38	agriculture	[86]
Bangladesh circa 1979	11.4	agriculture	[147]
Catalonia 1860	34.6	agriculture	[148]
Classical Greek	30.5	agriculture	[86]
Classical Greek	38	agriculture	[86]
Czechia 1850	39	agriculture	[149]
England Wales 1560	20	agriculture	[150]
England Wales 1600	17.4	agriculture	[150]
Europe 1500 CE	30	agriculture	[151]
Generic	26	agriculture	[152]
Han China	41	agriculture	[86]
Rome	9.2	agriculture	[153]
Rome	16.8	agriculture	[153]
Rome	38	agriculture	[86]
Sang Saeng	48	agriculture	[154]
Song China	45	agriculture	[86]
Trinket Island	39	agriculture	[155]
World 1820	19.2	agriculture	[156]
Generic	12	horticulture	[152]
Human-powered agriculture	9.5	horticulture	[157]
Generic	3.8	hunting-gathering	[157]
Generic	5	hunting-gathering	[152]
Western Eurasia 10,000 BCE	7.6	hunting-gathering	[86]
Western Eurasia 14,000 BCE	6.1	hunting-gathering	[86]

#### Sources for Fig 9

Fig 9A. Merges all sources from Fig 8. Fig 9B. The hierarchical despotism index *β* is estimated by matching empirical data to the best fit model iteration. *β* is chosen by minimizing the following error function:

$$\epsilon_{i}=|\text{log}E_{r}-\text{log}E_{m,i}\left|+\right|G_{r}-G_{m,i}|$$ (4)

*E*_*r*_ and *G*_*r*_ are energy use per capita and the Gini index of inequality (respectively) in the real-world society. *E*_*m*,*i*_ and *G*_*m*,*i*_ are energy use per capita and the Gini index of inequality (respectively) in the model iteration *i*. I assign real-world societies the model parameter *β*_*i*_ associated with the best-fit model iteration *i*.

### 7.2 Hierarchy model equations

This section provides technical details for the algorithm used to generate institutional hierarchies. Notation is shown in Table 2.

**Table 2:** Hierarchy model notation.

Symbol	Definition
*a*	span of control parameter 1
*b*	span of control parameter 2
*E*	employment
*h*	hierarchical level
*n*	number of hierarchical levels in an institution
*s*	span of control
*T*	total for institution
↓	round down to nearest integer
∏	product of a sequence of numbers
∑	sum of a sequence of numbers

#### 7.2.1 Generating the employment hierarchy

To generate the hierarchical structure of an institution, we begin by defining the span of control (*s*) as the ratio of employment (*E*) between two consecutive hierarchical levels (*h*), where *h* = 1 is the *bottom* hierarchical level. It simplifies later calculations if we define the span of control in level 1 as *s* = 1. This leads to the following piecewise function:

$$s_{h}\equiv \{\begin{array}{cc} \;\;1 & \text{if}\;\;h=1\\ \;\;\frac{E_{h}}{E_{h-1}} & \text{if}\;\;h\geq 2 \end{array}$$ (5)

The model assumes that the span of control is *not* constant; rather it increases *exponentially* with hierarchical level. I model the span of control as a function of hierarchical level (*s*_*h*_) with an exponential function, where *a* and *b* are free parameters:

$$s_{h}=\{\begin{array}{cc} \;\;1 & \text{if}\;\;h=1\\ \;\;a\cdot e^{bh} & \text{if}\;\;h\geq 2 \end{array}$$ (6)

As one moves up the hierarchy, employment in each consecutive level (*E*_*h*_) *decreases* by 1/*s*_*h*_. This yields Eq 7, a recursive method for calculating *E*_*h*_. Since we want employment to be *whole* numbers, we round down to the nearest integer (notated by ↓). By repeatedly substituting Eq 7 into itself, we can obtain a non-recursive formula (Eq 8). In product notation, Eq 8 can be written as Eq 9.

$$E_{h}=↓\frac{E_{h-1}}{s_{h}}\;\;\;\;\;\;\text{for}\;\;\;\;\;\;h>1$$ (7)

$$E_{h}=↓E_{1}\cdot \frac{1}{s_{2}}\cdot \frac{1}{s_{3}}\cdot \ldots \cdot \frac{1}{s_{h}}$$ (8)

$$E_{h}=↓E_{1}\prod_{i=1}^{h}\frac{1}{s_{i}}$$ (9)

Total employment in the whole institution (*E*_*T*_) is the sum of employment in all hierarchical levels. Defining *n* as the total number of hierarchical levels, we get Eq 10, which in summation notation, becomes Eq 11.

$$E_{T}=E_{1}+E_{2}+\ldots +E_{n}$$ (10)

$$E_{T}=\sum_{h=1}^{n}E_{h}$$ (11)

In practice, *n* is not known beforehand, so we define it using Eq 9. We progressively increase *h* until we reach a level of zero employment. The highest level *n* will be the hierarchical level directly *below* the first hierarchical level with zero employment:

$$n=\{h\;\;\;|\;\;\;E_{h}\geq 1\;\;\;\text{and}\;\;\;E_{h+1}=0\}$$ (12)

To summarize, the hierarchical employment structure of our model institution is determined by 3 free parameters: the span of control parameters *a* and *b*, and base-level employment *E*_1_. Code for this hierarchy generation algorithm can be found in the C++ header files hierarchy.h and exponents.h, located in the Supplementary Material [158].

#### 7.2.2 Calculating hierarchical power in the hierarchy model

I define an individual’s hierarchical power as one plus the number of subordinates (*S*) under their control:

P=1+S (13)

Because the hierarchy model simulates only the *aggregate* structure of institutions (employment by hierarchical level), hierarchical power is calculated as an *average* per rank. For hierarchical rank *h*, the average hierarchical power (${\bar{P}}_{h}$) is defined as the average number of subordinates (${\bar{S}}_{h}$) plus 1:

$${\bar{P}}_{h}=1+{\bar{S}}_{h}$$ (14)

Each individual with rank *h* is assigned the average power ${\bar{P}}_{h}$. The average number of subordinates ${\bar{S}}_{h}$ is equal to the sum of employment (*E*) in all subordinate levels, divided by employment in the level in question:

$${\bar{S}}_{h}\;=\sum_{i=1}^{h-1}\frac{E_{i}}{E_{h}}$$ (15)

**Fig 11:**
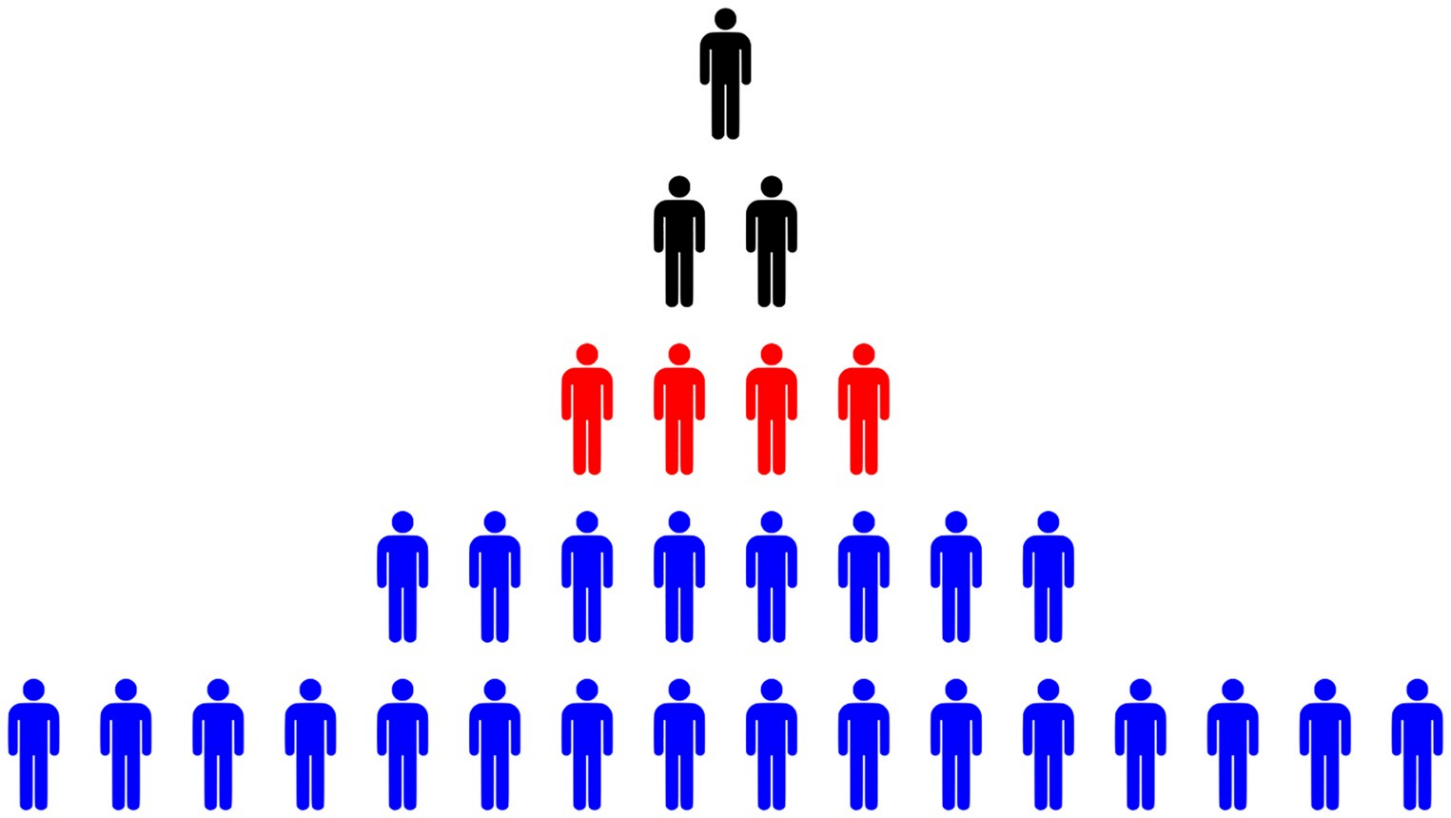
Calculating the average number of subordinates.

As an example, consider the hierarchy in Fig 11. The average number of subordinates below each individual in hierarchical level 3 (red) would be:

$${\bar{S}}_{3}\;=\;\frac{E_{1}+E_{2}}{E_{3}}\;=\;\frac{16+8}{4}=6$$ (16)

Therefore, these individuals would all be assigned a hierarchical power of 7.

### 7.3 Restricting model parameters

The model’s parameters are summarized in Table 3. My method for restricting these parameters is detailed below.

**Table 3:** Model parameters.

Parameter	Definition	Action	Scope
*α*	Institution size distribution exponent	Determines the skewness of the institution size distribution	—
*a*, *b*	Span of control parameters	Determines the shape of the institution hierarchy.	Identical for all institutions.
*E*_1_	Employment in base hierarchical level	Used to build the employment hierarchy from the bottom up. Determines total employment.	Specific to each firm.
*β*	Power-income exponent	Determines scaling relation between income and hierarchical power.	Identical for all institutions.
*σ*	Noise parameter	Used to add noise to the power-income relation. It is the scale parameter of a lognormal distribution	Identical for all institutions.

#### 7.3.1 Institution size distribution power-law exponent

Recent studies have found that firm size distributions in the United States [49] and other G7 countries [51] can be modeled accurately with a power law. A power law has the simple form shown in Eq 17, where the probability of observation *x* is inversely proportional to *x* raised to the exponent *α*:

$$p\left(x\right)\propto \frac{1}{x^{\alpha }}$$ (17)

The hierarchy model assumes that all human societies have power-law institution size distributions. The model simulates different societies by allowing the power-law exponent *α* to vary stochastically between different model iterations.

A characteristic property of power-law distributions is that as *α* approaches 2, the mean becomes *undefined*. In the present context, this means that the model can produce institution sizes that are extremely large—far beyond anything that exists in the real world. To deal with this difficulty, I *truncate* the power-law distribution at a maximum institution size of 2.3 million. This is the present size of Walmart, the largest firm that has ever existed.

Code for the discrete power-law random number generator can be found in the C++ header file rpld.h, located in the Supplementary Material [158]. This code is an adaptation of Collin Gillespie’s [144] discrete power-law generator found in the R poweRlaw package (which is, in turn, an adaptation of the algorithm outline by Clauset [159]).

#### 7.3.2 Span of Control Parameters

**Fig 12:**
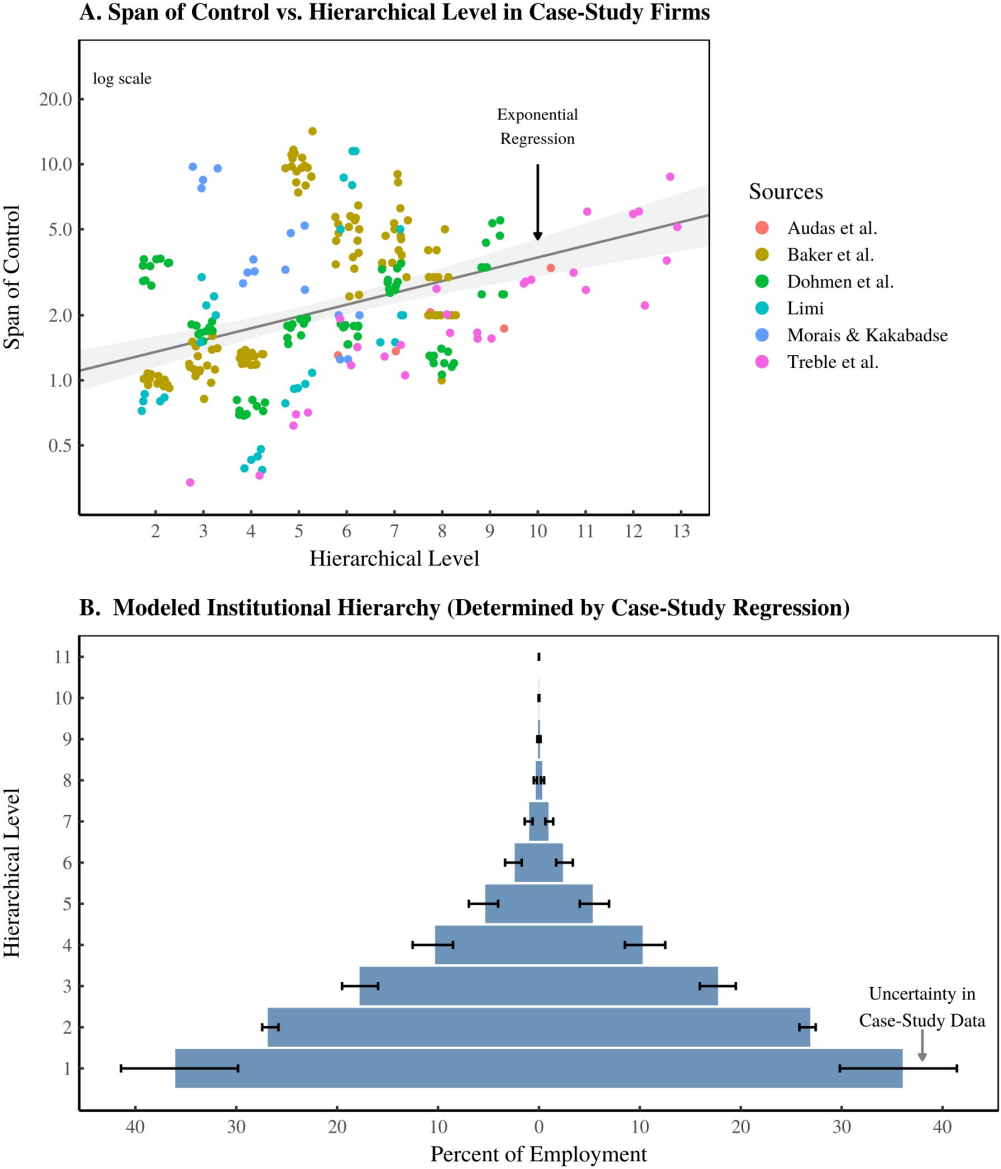
Idealized hierarchy implied by firm case studies. Panel A shows how the span of control varies with hierarchical level in case-study firms [57–62]. The span of control is the subordinate-to-superior ratio between adjacent hierarchical levels. The *x*-axis corresponds to the *upper* hierarchical level in each corresponding ratio. Case-study firms are indicated by color. Horizontal ‘jitter’ has been introduced to better visualize the data. The line indicates an exponential regression, with the grey region indicating the regression 95% confidence interval. Panel B shows the idealized firm hierarchy that is implied by the regression in Panel A. Error bars show the uncertainty in the hierarchical shape, calculated using a bootstrap resample of case-study data.

The parameters *a* and *b* together determine the shape of the model’s institutional hierarchies. These parameters are estimated from an exponential regression on firm case-study data (Fig 12A). The model assumes that these parameters are *constant* across all institutions. The resulting modeled hierarchy shape is shown in Fig 12B.

Because the case-study sample size is small, there is considerable uncertainty in the span of control parameters. I incorporate this uncertainty into the model using the *bootstrap* method [160], which involves repeatedly resampling the case-study data (with replacement) and then estimating the parameters *a* and *b* from this resample. I run the model many times, each time with *a* and *b* determined by a bootstrap resample of case-study data. The resulting variation in the shape of the model’s hierarchies is indicated by the error bars in Fig 12B. Code implementing this bootstrap can be found in the C++ header file boot_span.h located in the Supplementary Material [158].

#### 7.3.3 Base-level employment

Given span of control parameters *a* and *b*, each hierarchy is constructed from the *bottom* hierarchical level up. Thus, we must know base level employment. To get this value, I input a range of different base employment values into Eqs 6, 9, and 11 and calculate total employment for each value. The result is a discrete mapping relating base-level employment to total employment. I then use the C++ Armadillo interpolation function to linearly interpolate between these discrete values. This allows us to predict base level *E*_1_, given total employment *E*_*T*_. Code implementing this method can be found in the C++ header file base_fit.h, located in the Supplementary Material [158].

#### 7.3.4 Power-income exponent

The model assumes that income scales with hierarchical power as

$$\frac{I_{h}}{I_{1}}={\left(P_{h}\right)}^{\beta }\cdot \epsilon$$ (18)

where *I*_*h*_ is income in hierarchical level *h*, *I*_1_ is income in the base hierarchical level, *P* is hierarchical power, and *ϵ* is the stochastic noise factor.

**Fig 13:**
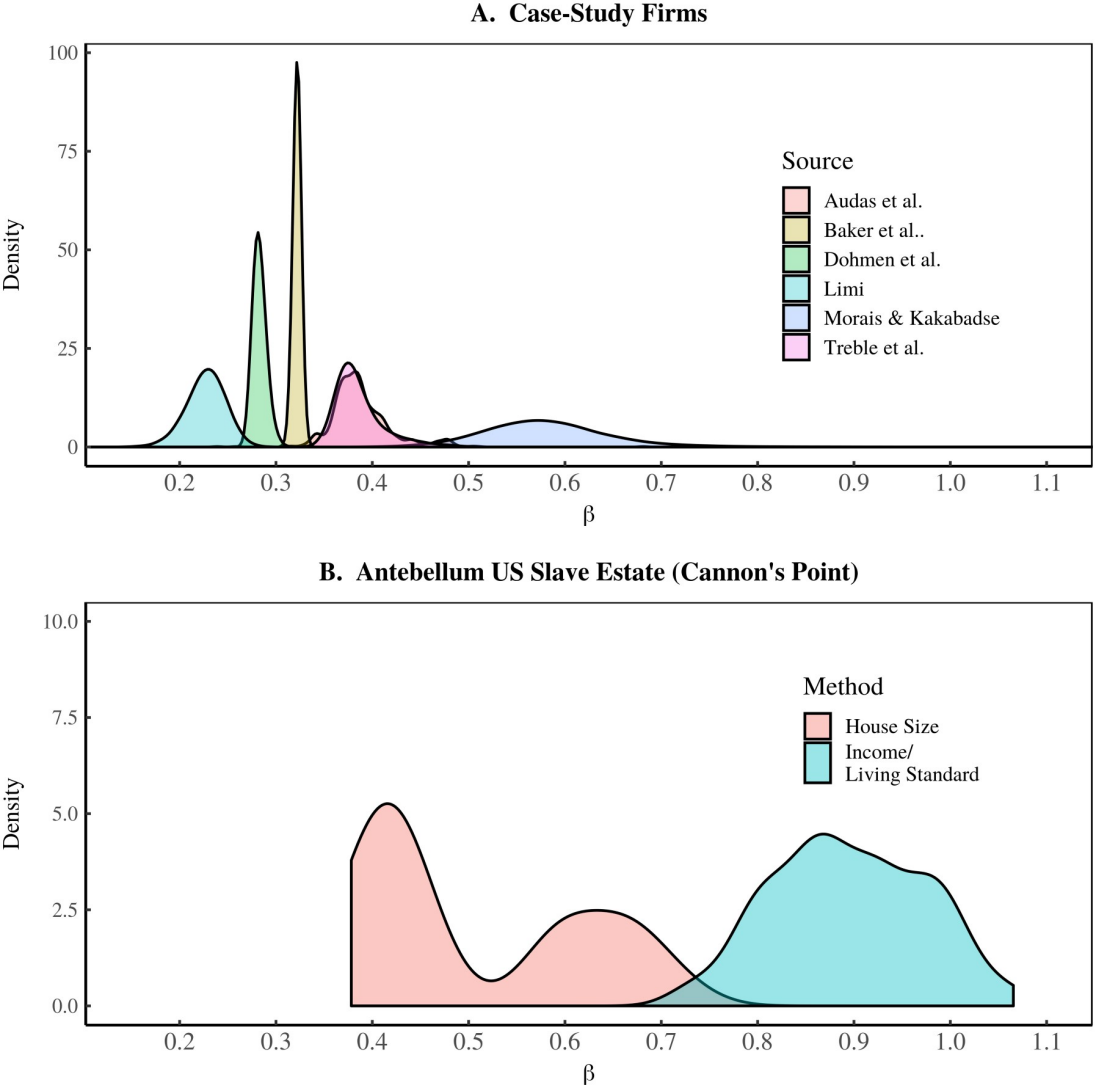
Probability distribution of *β* in case-study institutions. This figure shows the probability distribution of the parameter *β* in different case-study institutions. This parameter indicates the scaling behavior between income and hierarchical power: income ∝ (hierarchical power)^*β*^. Probabilities are determined using the bootstrap method. Panel A shows the *β* probability distribution for case-study firms [57–62]. Panel B shows the *β* probability distribution for a US slave estate (Cannon’s Point Plantation [161]). I show results for measuring inequality in terms of both house size and income.

To simulate variation between societies, I allow *β* to vary over different model iterations. I use two different data sources to determine a plausible range for this variation. The first is case-study data from modern firms [57–62]. I determine *β* from regressions on the data shown in Fig 4. For each case-study firm, I regress log(*I*_*h*_/*I*_1_) onto log*P*_*h*_. The slope of the relation is the estimate for *β*. I estimate the uncertainty in *β* using the bootstrap method [160]. I repeatedly resample case-study data and re-run the regression to estimate *β*. The resulting probability distribution of *β* is shown in Fig 13A for each case-study firm.

The second data source is a case study of a US slave estate—Cannon’s Point Plantation [161]. I estimate *β* from the living standard of the plantation owner relative to his slaves. For this estimate, we solve the power-income relation for *β*:

$$\beta =\frac{\text{log}(I_{h}/I_{1})}{\text{log}(P_{h})}$$ (19)

Although we do not know the hierarchical structure of the slave estate, we know that the owner sits on top of the hierarchy. All of the slaves are his subordinates. Therefore the number of slaves (*n*_slave_) gives us a rough estimate for the owner’s hierarchical power:

$$P_{\text{owner}}\approx 1+n_{\text{slave}}$$ (20)

If we know the living standard of the owner (*I*_owner_) and slaves (*I*_slave_), we can combine Eqs 19 and 20 to get a rough estimate for *β*:

$$\beta \approx \frac{\text{log}(I_{\text{owner}}/I_{\text{slave}})}{\text{log}(1+n_{\text{slave}})}$$ (21)

The living standard of the owner is equal to his income. But slaves have no income, so we must use another method to estimate their living standards. One way is to use the slave expenses paid by the owner. Another method is to compare the owner and slaves in terms of house size. The results for both methods are shown in Fig 13B. Again, I use the bootstrap technique to investigate the plausible range of *β* that is implied by the Cannon’s Point data. I sample different values for the owner’s income, the slaves’ income (living standard), and the number of slaves and put them repeatedly into Eq 21.

As we would expect, the resulting *β* for our slave estate is far higher than in our case-study firms. In a slave regime, the evidence suggests that *β* could approach 1. To put this in perspective, this means income scales *linearly* with hierarchical power. If this were the case in industrial societies, the CEO of Walmart would earn 2 million times that of an entry-level worker. Nothing like this exists in industrial societies—for good reason. They are not based on slavery. But slavery was ubiquitous in human history, so we need to allow for its existence in our model.

Based on the case-study data in Fig 13, I allow *β* to vary over the range 0.2 ≤ *β* ≤ 1.

#### 7.3.5 Power-income noise factor

Noise (*ϵ*) in the power-income relation is modeled with a lognormal random variate with dispersion determined by the parameter *σ*:

ϵ∼lnN(σ) (22)

**Fig 14:**
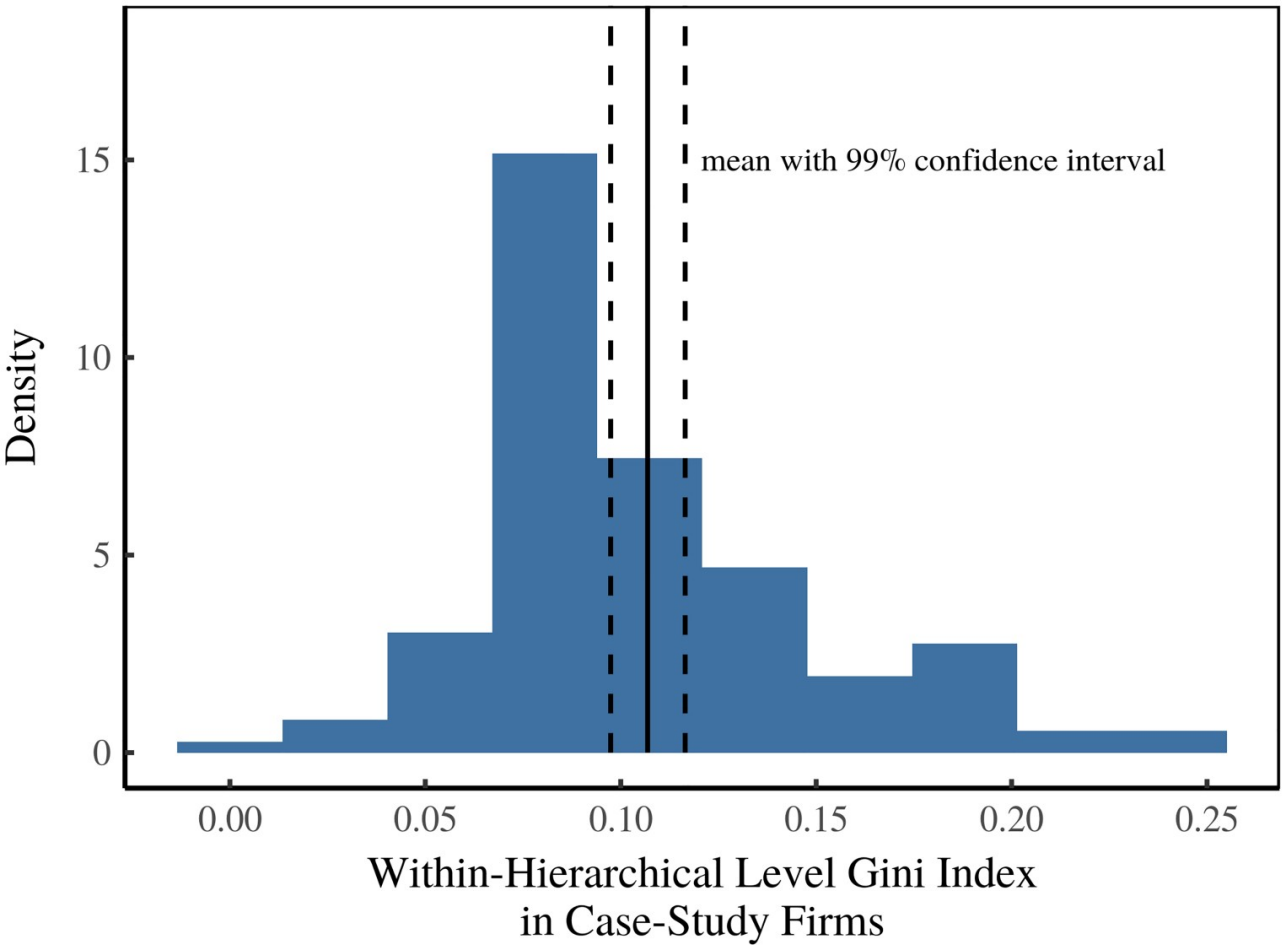
Determining the power-income ‘Noise’ parameter. This figure shows the distribution of income dispersion within hierarchical levels of case-study firms [57–62, 162], measured using the Gini index. The mean of this distribution (with associated uncertainty) is used to set the power-income noise parameter *σ*. When not reported directly (or calculable from raw data), the within-hierarchical level Gini index is estimated from reported summary statistics in case studies.

The noise factor reproduces the average within-hierarchical level income dispersion in case-study firms [57–62]. The distribution of within-hierarchical level income dispersion is shown in Fig 14. To determine *σ*, we first calculate the mean Gini index ($\bar{G}$) of the case-study data shown in Fig 14. We then calculate *σ* using:

$$\sigma =2\cdot {\text{erf}}^{-1}\left(\bar{G}\right)$$ (23)

This equation is derived from the definition of the Gini index of a lognormal distribution: *G* = erf(*σ*/2). To incorporate uncertainty in the case-study data, each model iteration uses a different bootstrap resample to calculate $\bar{G}$. Code implementing this method can be found in the C++ header file boot_sigma.h, located in the Supplementary Material [158].

#### 7.3.6 Estimating energy use from average institution size

The energy-hierarchy-inequality model assumes that energy use *E*_*pc*_ is proportional to average institution size *I*:

$$E_{pc}=c_{1}{\bar{I}}^{c_{2}}$$ (24)

The parameters *c*_1_ and *c*_2_ are determined from regressions on the international firm data shown in Fig 1A.

## Supporting information

S1 Fig Distribution of slave ownership in the US South in 1860. The blue line shows the distribution of slave ownership in the US South. ‘Steps’ indicate the bins in the original data. The red line shows the best-fit power-law distribution, which has an exponent *α* = 2.7. The shaded region indicates the range of uncertainty for a sample of 1 million. Slave-estate size roughly follows a power-law distribution. Data is from [163], as reported in [164]. The best-fit power law is determined using the methods in [165].
(TIFF)
