# Supplementary figures and images for: Energy, hierarchy and the origin of inequality
Source: PLoS One. 2019 Apr 24;14(4):e0215692. doi: 10.1371/journal.pone.0215692 (PMC6481848; doi:10.1371/journal.pone.0215692)

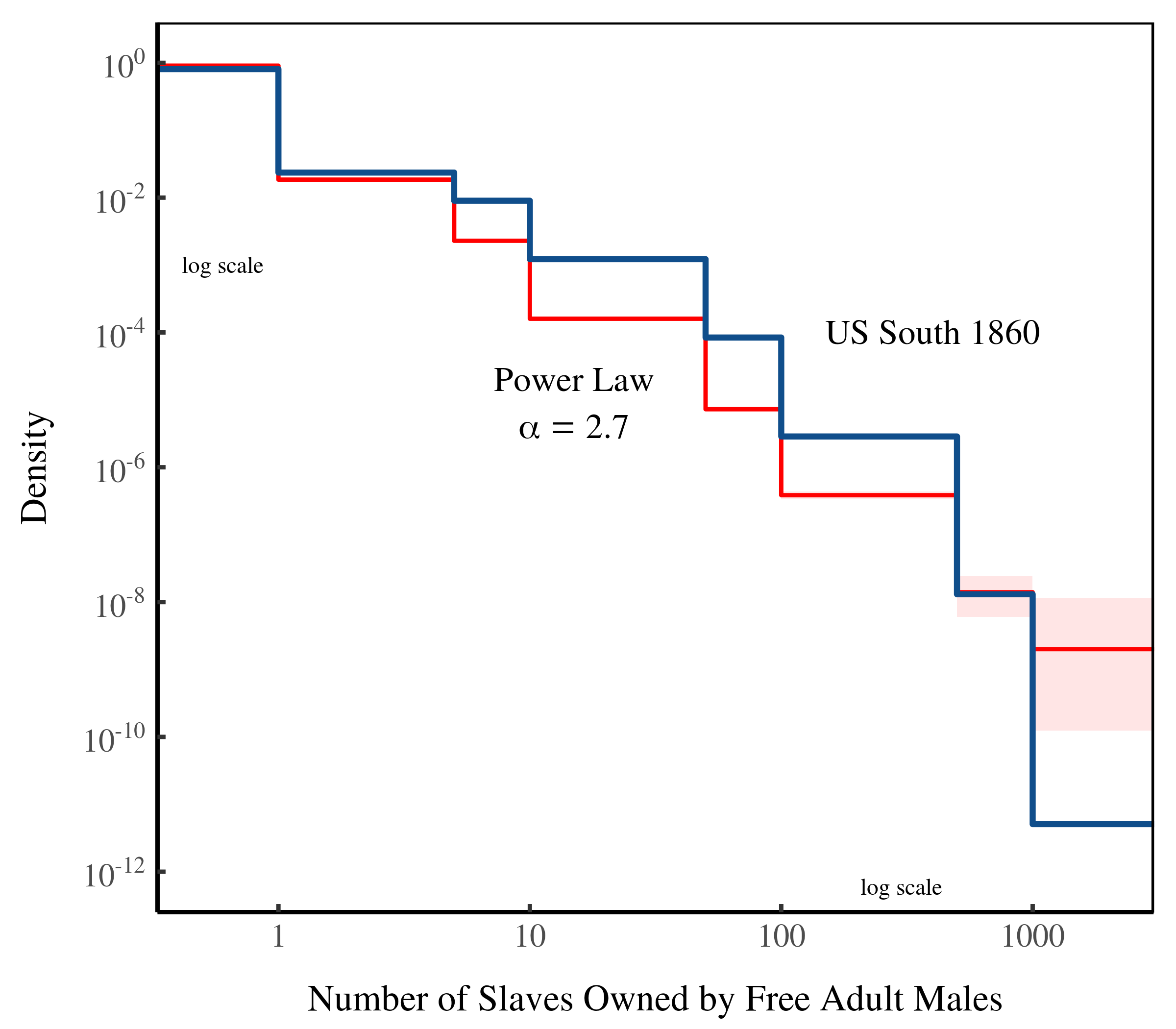

Supplement: S1 Fig — The blue line shows the distribution of slave ownership in the US South. ‘Steps’ indicate the bins in the original data. The red line shows the best-fit power-law distribution, which has an exponent α = 2.7. The shaded region indicates the range of uncertainty for a sample of 1 million. Slave-estate size roughly follows a power-law distribution. Data is from [163], as reported in [164]. The best-fit power law is determined using the methods in [165]. (TIFF) [file pone.0215692.s001.tiff]
